# Bodily ownership modulation in defensive responses: physiological evidence in brain-damaged patients with pathological embodiment of other’s body parts

**DOI:** 10.1038/srep27737

**Published:** 2016-06-13

**Authors:** C. Fossataro, P. Gindri, T. Mezzanato, L. Pia, F. Garbarini

**Affiliations:** 1 SAMBA – SpAtial, Motor & Bodily Awareness – Research Group, Psychology Department, University of Turin, Turin, Italy; 2 San Camillo Hospital of Turin, Turin, Italy; 3 Ausiliatrice - Don Gnocchi Hospital of Turin, Turin, Italy; 4 Neuroscience Institute of Turin (NIT), University of Turin, Italy

## Abstract

Do conscious beliefs about the body affect defensive mechanisms within the body? To answer this question we took advantage from a monothematic delusion of bodily ownership, in which brain-damaged patients misidentify alien limbs as their own. We investigated whether the delusional belief that an alien hand is their own hand modulates a subcortical defensive response, such as the hand-blink reflex. The blink, dramatically increases when the threated hand is inside the defensive peripersonal-space of the face. In our between-subjects design, including patients and controls, the threat was brought near the face either by the own hand or by another person’s hand. Our results show an ownership-dependent modulation of the defensive response. In controls, as well as in the patients’ intact-side, the response enhancement is significantly greater when the threat was brought near the face by the own than by the alien hand. Crucially, in the patients’ affected-side (where the pathological embodiment occurs), the alien (embodied) hand elicited a response enhancement comparable to that found when the threat is brought near the face by the real hand. These findings suggest the existence of a mutual interaction between our conscious beliefs about the body and the physiological mechanisms within the body.

Converging multidisciplinary approaches described the sense of bodily ownership (i.e., the belief that a specific body part belongs to one’s own body) as a fundamental component of the self-consciousness^1^,^2^,^3^. In normal conditions, it seems immediate and even obvious. However, both experimental manipulations in healthy subjects, such as the rubber hand illusion (RHI)^4^, and pathological conditions, after brain damages, suggest that the sense of bodily ownership can be selectively altered. During the RHI procedure, watching a rubber hand being stroked, while one’s unseen hand is stroked synchronously, can lead to qualitative (the reported sense of ownership over the rubber hand) and quantitative (a shift in the perceived position of the real hand towards the fake hand) modulation of the sense of bodily ownership, so that the rubber hand is embodied and the real hand is subjected to a sort of disembodiment^5^.

From a neuropsychological perspective, pathological conditions, where the sense of bodily ownership is dramatically impaired, can be assumed as a model to understand the normal construction of self-awareness. After brain damages, the more frequent body-related disorder is characterized by a sense of disownership, which is the delusional belief that the contralesional limbs do not belong to one’s own body but to another person (a disturbance called somatoparaphrenia^6^,^7^,^8^). The opposite behavior has been recently discovered in patients who misidentify other people’s limbs as their own, reporting a monothematic delusion of bodily ownership. We observed this behavior in a sample of hemiplegic and/or hemianesthesic patients. While they did not explicitly deny that their contralesional limbs belonged to themselves (as in the somatoparaphrenia), they claimed that the examiner’s hand was their own, whenever it was positioned on the table, next to their real hand. This delusion of ownership, although resembling the RHI, was spontaneous and not induced by any experimental procedure. Patients suffering from this disorder treat and care for the examiner’s hand as if it was their own, showing a consistent embodiment of an alien body part. Thus, we called them E+ patients. It is interesting to note that, according to specific constraints similar to those operating in the RHI, the pathological embodiment occurs only when the alien hand is perceived in egocentric coordinates and it is aligned with the patients’ contralesional shoulder, in a coherent position with respect to the patients’ body schema (i.e., our unconscious and dynamic body representation^9^). If the alien hand is presented in different positions or in the intact body-side, the pathological embodiment does not occur and patients correctly discriminate the own from the alien hand^10^,^11^,^12^,^13^,^14^ (see also a video at http://youtu.be/uKTwAybB758). This evidence suggest that, in both RHI and E+ patients, in order for the embodiment to occur, bottom-up multisensory integration processes^15^,^16^ are not sufficient, but, instead, top-down preexisting body representations, containing information about what can be potentially included in my own body according to specific constraints, are necessary^17^,^18^.

In the present study, we asked whether a top-down process, such as this delusional belief that an alien hand is a part of the own body, could modulate a bottom-up mechanism, such as the eye blink elicited when the hand receive a painful stimulation (Hand Blink Reflex, HBR). The HBR is considered a subcortical defensive response, entirely mediated at the brainstem level^19^. It is known that this response is modulated by the hand position in space: indeed, it dramatically increases when the threated hand is located close to the face, inside the defensive peripersonal space (DPPS - a vital safety margin surrounding the body)^20^,^21^,^22^,^23^,^24^,^25^,^26^. Here, we investigated whether, in E+ patients, the magnitude of the HBR is enhanced irrespective of whether the threat is brought near the face by the own or the alien (embodied) hand (see Fig. 1A and Materials and Methods). Note that, because of the hemiplegia, in the patients’ affected side the hand postural manipulation (far from the face; near to the face) was passively performed by means of the experimenter’s help. In a preliminary study (see details in Materials and Methods), we verified that the passive movement is not a significant factor by itself in the HBR modulation. Furthermore, to control for the effect induced by sensory-motor deficits on our task, we included in the pathological sample a brain-damaged patient with primary deficits similar to those showing by E+ patients, but without pathological embodiment (hereinafter, E− patient). See patients’ characteristics details in Materials and Methods and in Table 1.

We know from the E+ patients clinical evaluation^11^,^12^,^13^,^14^ that, when the alien limb (either right or left examiner’s limb) is positioned in the intact body-side, the patients correctly recognize it as belonging to another person. Thus, what is crucial for the pathological embodiment to occur is the position of the alien limb in space. Accordingly, the critical aspect of the present study is that, in the E+ patients’ affected body-side, where the pathological embodiment occurred, the patients were convinced that the alien hand, undertaking the postural manipulation (up to or down from the face), was their own hand. On the contrary, in the E+ patients’ intact body-side, where the pathological embodiment did not occur, patients correctly discriminated between the own and the alien hand. As a consequence, in the E+ patients’ intact body-side, as well as in healthy controls and in the E− patient with normal sense of bodily ownership, we could expect to find a difference in the HBR modulation, depending on the hand (own or alien) performing the postural manipulation. In contrast, when the sense of bodily ownership is dramatically altered, as in the E+ patients’ affected body-side, we could expect to find a similar HBR modulation, irrespective of whether the postural manipulation was performed by the own or by the alien (embodied) hand. Thus, in E+ patients, a crucial difference between the alien condition in the intact and in the affected body-side was expected.

## Results

**Figure 1:**
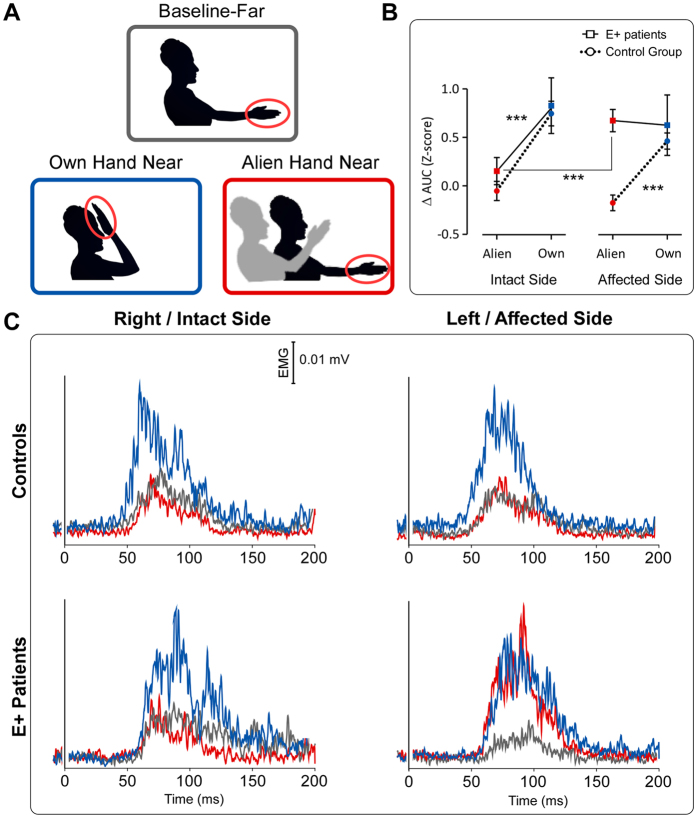
Conditions, group-average waveforms and results. (**A**) Experimental Conditions. **(B**) ANOVA results. Graphs show the AUC HBR mean values (Z-scores) with standard errors for the own-hand and the alien-hand condition, expressed as difference with respect to the baseline, in both Controls group (N = 10) and E+ patients (N = 5). ***P < 0.0001. (**C**) Controls’ (***Top***) and E+ patients’ (***Bottom***) Group-average, rectified HBR waveforms; x-axis, Time (ms); y-axis EMG activity (mV) in all three conditions, in both the intact/right and the affected/left body-side.

We extracted the area under the curve (AUC) of the average HBR waveform and we normalized it in z-scores. As dependent variable, we expressed the AUC data for the “own-hand-near” and the “alien-hand-near” conditions as a difference respect to the “baseline-far” condition (see details in Materials and Methods). The obtained values were entered in a mixed factors 2 × 2 × 2 ANOVA. The results showed a significant effect of ‘hand ownership’ (F_(1,13)_ = 39.28, p = 0.00003), suggesting that, overall, the response enhancement in the near position (close to the face) was greater for the own than for the alien hand. More crucial to the present study, we found a significant interaction ‘body-side*ownership*group’ (F_(1,13)_ = 11.21, p = 0.0052) (Fig. 1B). This suggests that, in controls, a similar ownership modulation (i.e. greater responses for the own- than for the alien-hand) was present in both the right and the left body-side (p < 0.0001 for each comparison). On the contrary, in the E+ patients, a significant difference in the ownership modulation was found, depending on the body-side where the postural manipulation was performed. In the E+ patients’ intact body-side, as in controls, greater responses was elicited by the own than by the alien hand (p = 0.00003); in the E+ patients’ affected body-side, no difference between the own and the alien hand was found (p = 0.94). Note that the responses in the affected body-side, for both the own and the alien hand, were comparable to the responses elicited by the own hand in the intact body-side. According to our predictions, a significant difference between the alien (embodied) hand in the affected body-side and the alien (not embodied) hand in the intact body-side was found (p = 0.00002). For both groups (controls and E+ patients), the average HBR waveforms for each condition are shown in Fig. 1C.

Crawford’s tests were used to investigate the difference between E− patient and both E+ patients and controls in the HBR enhancement with respect to the baseline (far position) in all the experimental conditions (alien-hand-near and own-hand-near in both intact and affected body-side). Crawford’s tests (two tailed) did not found any significant difference between E− patients and control group. Crucially, it revealed a significant difference between E− patient and E+ group only in the alien-hand-near condition of the affected body side (t = −4.086; p = 0.01; ZCC = −4.47). On the contrary, no significant difference was found in the own-hand-near condition of the affected body side and in the alien-hand-near and own-hand-near condition of the intact body side.

**Figure 2:**
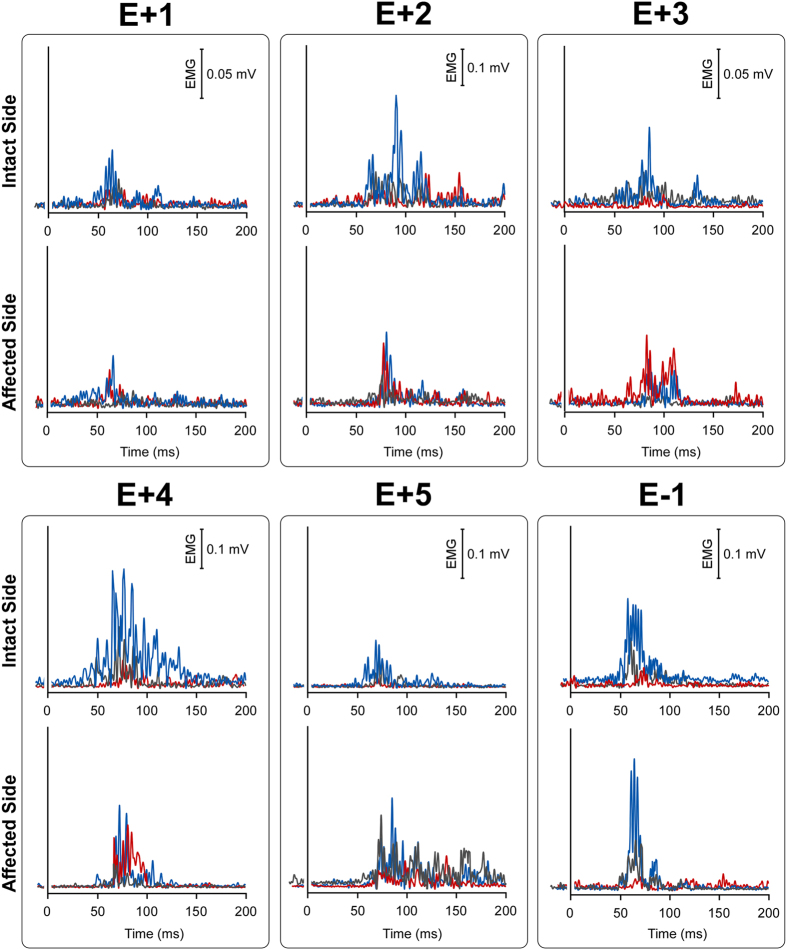
Single subjects HBR waveforms. E+ patients and E− patient single subjects rectified HBR waveforms; x-axis, Time (ms); y-axis EMG activity (mV) in all conditions, both in the intact/right and the affected/left body-side.

For each E+ and E− patient, single-subject waveforms are shown in Fig. 2. Note that, in the affected body-side, for each E+ patient, the red (alien-hand-near) and the blue (own-hand-near) curves show a similar pattern, being always greater with respect to the baseline in gray. On the contrary, for the E− patient, only the blue curve is greater than the gray one, in both intact and affected body-side.

## Discussion

The present study is related to the sense of bodily ownership, that has been described, by converging multidisciplinary approaches, as a fundamental component of the self-consciousness^1^,^2^,^3^,^15^,^28^,^29^,^30^,^31^,^32^,^33^,^34^,^35^,^36^. In particular, we sought for evidence that a specific bottom-up mechanism, such as the HBR, can be affected by a top-down process, such as the sense of bodily ownership. Our results show a compelling body ownership-dependent modulation of the defensive system. In healthy controls, as well as in the E− patient and in the E+ patients’ intact body-side, where the sense of bodily ownership is spared, the presence of an alien hand inside the subject’s DPPS is not sufficient to induce an HBR enhancement comparable to that found for the own hand. On the contrary, in the E+ patients’ affected side, where the sense of bodily ownership is dramatically altered, the magnitude of the HBR is enhanced irrespective of whether the threat is brought near the face by the own or the alien (embedded) hand.

The data discussed here confirm previous evidences, both in the physiological and in the neuropsychological domain, also adding some important new findings. In the physiological domain, our results are in keeping with the previously described “hand position” effect, suggesting that the HBR is significantly enhanced, by approximately a factor of two, when one’s own stimulated hand enters the DPPS of the face^20^,^21^,^22^,^26^. As suggested by Sambo and colleagues, such enhancement may result from the modulation of the excitability of brainstem circuits mediating the HBR by associative cortical areas involved in the representation of peripersonal space and in the detection of potentially dangerous stimuli near the face^25^. We also confirm (see Preliminary analysis and preliminary results in Materials and Methods) that the HBR increase was present when the stimuli were delivered to both the ipsilateral and the contralateral hand with respect to the body-side where the postural manipulation was performed, although for the ipsilateral (moving) hand the HBR enhancement in the near position was greater^20^. Finally, the patients’ results (see Preliminary analysis and preliminary results in Materials and Methods) confirm previous studies^37^ showing a suppression of the R2 blink reflex after stimulation of the affected side in brain-damaged patients with hemiplegia, that can be likely attributed to the decreased cortical-reticular drive. To this reason, in patients, only responses coming from the ipsilesional (intact) body-side were considered for analyses (see details in Materials and Methods).

Two new physiological findings, related to the HBR modulation, were found in the present study. The first (negative) result shows that the volitional component of the movement, through which the subject keep the hand far-from or near-to the face, is not able to modulate the strength of the HBR enhancement. Indeed, in the healthy subjects involved in the preliminary study on the passive condition (see Preliminary analysis and preliminary results in Materials and Methods), as well as in the hemiplegic patients tested in the main experiment, we found that keeping the own hand actively or passively close to the face induce a similar modulation in the HBR magnitude. The second (positive) result shows that the bodily ownership plays a crucial role in modulating the strength of the HBR enhancement. Indeed, in healthy controls and in the E+ patients’ intact body-side, the HBR enhancement is significantly greater when the own hand enters the DPPS, with respect to when the alien hand is placed inside the DPPS, while one’s own hand is placed outside (similar results were found in a different context, where the empathy modulation of defensive responses were investigated^26^). This means that simply looking at an alien hand, located close to the own face, is not sufficient to induce an HBR enhancement comparable to that found for the own hand. In this case, the information coming from the own stimulated hand, kept far from the face in a “safe” position, is prioritized.

In the neuropsychological domain, we confirm previous studies suggesting that the pathological embodiment occurs not only with a static alien hand, but also with a moving alien hand^11^,^13^. Accordingly, when the alien hand performed the postural manipulation in the affected body-side, the E+ patients reported to perform the postural manipulation with their own hand (see Embodiment evaluation during the task in Materials and Methods). We are also in keeping with a previous finding showing that, in the somatosensory domain, when painful stimuli are delivered to the alien embodied hand, E+ patients refer to feel pain on it^14^ and show an altered skin conductance response (SCR)^12^. Accordingly, in the affected body-side, E+ patients tested here reported to feel the electrical stimuli on the alien embodied hand, keeping close to their face (see Procedures in Materials and Methods).

By combining physiological and neuropsychological approaches, we added an important new finding in both domains: a pathological belief about the body modulates a subcortical defensive response, entirely mediated by a neural circuit at the brainstem level (i.e., the HBR). What we found is that, in the E+ patients’ affected body-side, the HBR enhancement, typically described when the own stimulated hand enters the DPPS of the face, is also present (with a similar extent) when the alien hand is placed inside the DPPS. During the experiment, E+ patients were convinced that the alien hand, undertaken the postural manipulation, was their own, and this induced a “non-veridical bodily experience” that the own hand was located close to their face. This, in turn, was able to produce a HBR enhancement comparable to that found for the own hand. Alternatively, we could explain this result referring to the E+ patients’ sensory-motor deficits (see Table 1), that can prevent the patients to detect the information coming from the own stimulated hand (located in a “safe” position, far from the face). However, the control E− patient, with sensory-motor deficits comparable to those present in E+ patients (see Table 1), did not show the HBR enhancement for the alien hand. This means that what is crucial in order for the HBR enhancement to occur is not the presence of primary deficits, but the sense of bodily ownership, intact in the E− patient and altered in the E+ patients.

The present findings have implications for our understanding of the relationship between explicit (conscious) bodily representation and implicit (unconscious) physiological reaction of potential threats directed toward the own body. Previous studies investigated this issue in both healthy subjects and brain-damaged patients. Armel and Ramachandran’s pioneering study^15^ showed that, during the RHI, the increased feeling of ownership over the fake hand correlates with the level of arousal responses (measured by using SCR) for sudden threats directed to the fake hand^38^,^39^. However, some studies, always manipulating the sense of bodily ownership in healthy subjects, showed that an interplay between distinct conscious and unconscious mechanisms may contribute to the detection of threatening approaching stimuli. Tieri and colleagues^40^ showed that the conscious embodiment of an artificial limb requires a natural looking visual body appearance, while implicit reactivity to threat, as measured by using SCR, may only require physical body continuity, even if the visual body appearance is not natural. Romano and colleagues^41^ showed that, although the strength of illusory self-identification with an avatar significantly predicts the analgesic effect induced by observing the embodied avatar’s hand while painful stimuli were delivered to the observers’ hand, this implicit physiological reduced response is not transferred in an aware reduced experience of pain. In all these studies in healthy subjects, the “illusory” sense of bodily ownership is usually assessed through ad hoc scales^4^,^42^,^43^ and the subjective ratings were used to investigate correlations with the physiological measure. However, the crucial difference between the experience induced by the RHI in healthy subjects and the pathological delusion of ownership, shown by E+ patients, is that in the former the subjects are always aware of the illusory nature of the experience and they can rate it, while the latter produces an impenetrable false belief over the hand ownership, only allowing a dichotomous choice (yes, it is my hand; no, it is not my hand). Thus, in E+ patients, no correlations between the extent of the delusion of ownership and the HBR amplitude could be directly investigated. However, the present data suggest that explicit (conscious) body representation are tight linked to implicit (unconscious) physiological reaction of potential threats directed toward the own body: in the patients’ affected-side, when E+ patients explicitly reported that the alien hand performing the postural manipulation (far/near the face) was their own hand, we found a HBR enhancement comparable to that found for the real hand. These results are also in keeping with previous findings in body-related disorders after brain damage. In the delusion of ownership, shown by E+ patients, an increased SCR was found when the threats were directed toward the alien (embodied) hand, that the patients explicitly reported as belonging to themselves^12^. Complementary results were found in somatoparaphrenic patients, showing a reduced SCR when the threats were directed toward the own (disembodied) hand, that the patients explicitly reported as belonging to another person^8^.

**Figure 3:**
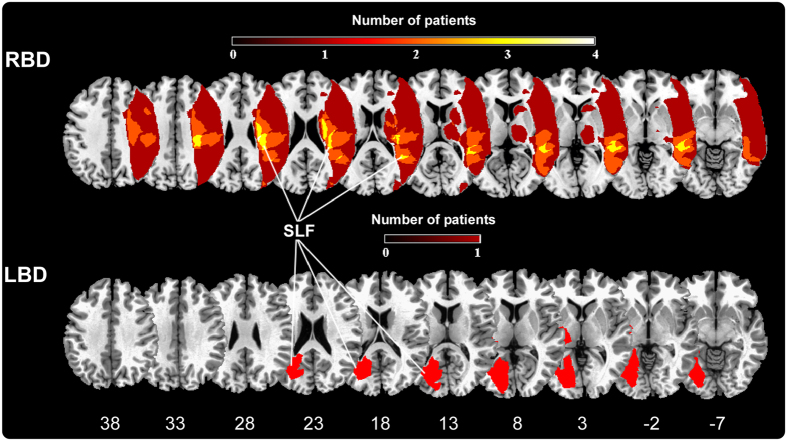
Overlay of lesion plots. Overlay of regional lesion plots of the E+ patients group. In RBD group the frequency is represented trough a color scale ranging from black (lesion in one patient) to yellow (lesion in 4 patients). Superior longitudinal fasciculus (SLF) is the structure more frequently damaged in our E+ group, is displayed in warm colors, from dark red to white. MNI coordinates of each transverse section are reported.

From an anatomical point of view, although this phenomenon seems to be more frequently associated to right-brain lesions, in the present E+ patients sample, as well as in a previous study^13^, we described a left brain-damaged case with right limb embodiment (E+1). This suggests the presence of a right hemisphere dominance for the building of the sense of body ownership rather than an absolute lateralization of this function in the right hemisphere^17^. According to previous studies^11^,^13^,^14^, the lesion mapping (see Fig. 3) shows that the pathological embodiment is mainly related to a subcortical involvement of the periventricular white matter. This lesion pattern is also consistent with the one identified for being responsible for somatoparaphrenia^7^, thus suggesting a common locus for the two complementary bodily awareness disorders. Particularly, the lesion overlay plotted in Fig. 3, shows a specific involvement of the superior longitudinal fasciculus (SLF), known to connect the frontal and parietal cortex. Within the bodily ownership literature, converging evidence showed that the feeling that our body belongs to us presumably depends on multisensory integration processes arising within a fronto-parietal network, where sensory inputs coming from different modalities are realigned in a unique reference frame^2^,^3^,^36^. Within this network, the ventral premotor cortex seems to play a crucial role, both in monkeys^44^ and in humans^16^,^45^, and a damage to the pathways connecting premotor cortex, prefrontal cortex and parietal cortex results in an inability to experience illusory hand ownership during the RHI^46^. Although the present anatomical result needs further investigation on a larger sample of patient’s lesion studies, we can speculate that a fronto-parietal disconnection, due to the SFL lesion, induces the altered bodily ownership shown by E+ patients and that, in turn, this exerts a top-down modulation on the subcortical circuit mediating the HBR.

The key finding of this study is that an alien hand, that becomes a part of the own body, is able to elicit defensive responses advantageous for survival, typically triggered when the threat is brought near the face by the real hand. These results, showing that the vulnerability of the sense of bodily ownership is accompanied by a coherent modulation of the defensive system, have important implications for our understanding of the human bodily awareness. They suggest the existence, in the normal functioning of the human brain, of a mutual interaction between our conscious beliefs about the body and the physiological mechanisms within the body.

## Materials and Methods

### Patients’ recruitment and participants

During the period between October 2013 and June 2015 eighteen brain-damaged patients of cerebrovascular origin, with contra-lesional upper limb sensory-motor deficits, were recruited at the “San Camillo” Hospital (Turin, Italy) and at the “Maria Ausiliatrice” Hospital (Turin, Italy). All patients were assessed using common neuropsychological tests: general cognitive tests (Montreal Cognitive Assessment – MOCA or Mini Mental State Evaluation - MMSE); visual field exam, with the score ranking from 0 to 3; assessment of the contralesional upper limb hemiplegia, as reported by the responsible neurologist and confirmed by a motor impairment examination carried out according to a clinical protocol^47^,^48^, with the score ranking from 0 to 3 ; assessment of anosognosia for hemiplegia with the score ranking from 0 to 3, assessment of hemianesthesia, with the score ranking from 0 to 3; assessment of anosognosia hemianesthesia, with the score ranking from 0 to 3; tests for neglect (Behavioral Inattention Test - BIT – conventional and behavioral subtests; DILLER) and for personal neglect (FLUFF). Patients were also evaluated for somatoparaphrenia^49^. See Table 1 for details. Exclusion criteria were: 1) previous neurological or psychiatric history; 2) severe general cognitive impairment; 3) visual field deficits. In order to include patients in the experimental group, we tested them with an ad hoc protocol devised to assess the presence/absence of pathological embodiment, proposed in previous studies^11^,^12^,^13^,^14^,^50^. According to this evaluation, nine out of eighteen patients were selected for the study. Patients’ brain lesions, as documented by clinical CT or MRI scans, are consistent with those described in previous studies^11^,^12^,^13^,^14^,^50^. Lesions were mapped onto the MNI stereotactic space with standard MRI volume (voxels of 1 mm^3^) through a computerized technique. Image manipulations were obtained with the software MRIcron^51^. Firstly, the MNI template was rotated on coronal, sagittal and horizontal planes according to the patient’s scan angle. Secondly, a skilled rater (LP) manually mapped the lesion onto each correspondent template slice, whereas a second skilled rater (CF) double-checked for the accuracy of the tracings for each patient. Thirdly, the maps were back rotated into the standard space. Grey matter involvement was obtained by superimposing the Anatomical Labelling map template AAL^52^ and the JHU-white matter template^53^ which categorize the distributions of digital images onto stereotactic space. The involved brain structures are summarized as following: **E+1**. Left-hemisphere lesions, involving: hippocampus, amygdala, middle temporal pole, parahippocampal gyrus, fusiform gyrus, heschl gyrus, temporo-parietal periventricular white matter, including superior longitudinal fasciculus (SLF). **E+2.** Right-hemisphere lesions, involving: Thalamus and sub-cortical white matter including posterior limb of internal capsule, retrolenticular part of internal capsule, superior corona radiata **E+3** = Right-hemisphere lesions, involving: supramarginal gyrus, middle temporal pole, heschl gyrus, uncinate fasciculus, cingulum (hippocampus), posterior thalamus, retrolenticular part of internal capsule, putamen, sagittal stratum and temporo-parietal periventricular white matter, including SLF. **E+4** = Right-hemisphere lesions, involving: precentral gyrus, inferior frontal operculum, inferior frontal gyrus (triangular part), inferior frontal gyrus (orbital part), rolandic operculum, insula, postcentral gyrus, supramarginal gyrus, angular gyrus, heschl gyrus, suoperioir temporal gyrus, temporal pole (superior part), middle temporal gyrus. fronto-temporo-parietal periventricular white matter, including SLF. **E+5** = Right-hemisphere lesions, involving: middle frontal gyrus and fronto-parietal periventricular white matter, including SLF and superior corona radiate. Finally, we created a lesions overlap. Grey and white matter regions involvement were obtained by superimposing the Anatomical Labeling map template AAL^52^ and the JHU-white matter template^53^. The overlay plot, showing an involvement of the SLF, is represented in Fig. 3.

Five out of nine E+ patients (3 females, mean age ± standard deviation: 63.4 ± 9.68) showing a reproducible HBR (i.e., observed in five consecutive trials) were involved in the experiment, four right brain-damaged patients showing left-limb embodiment and one left brain-damaged patient showing right-limb embodiment. Additionally, one E− patient, with sensory-motor deficits similar to those shown by E+ patients (See Table 1), was involved as control patient. Note that, during the task, the presence/absence of the pathological embodiment was also tested with an on-line embodiment evaluation (see Embodiment evaluation during the task). Finally, ten aged-matched healthy subjects (7 females, mean age ± standard deviation: 54.9 ± 8.06) were engaged in the experiment as control group. All participants were naive to the experimental procedure and to the purpose of the study and provide written informed consent to participate in the study. In accordance with the Declaration of Helsinki (BMJ 1991; 302: 1194) all the experimental procedures were approved by the Ethical Committee of the ASL TO 1 of Turin.

### Stimulation and recording

Electromyographic activity (EMG) was recorded from the orbicularis oculi muscle bilaterally, using two pairs of bipolar surface electrodes with the active electrode over the mid lower eyelid and the reference electrode laterally to the outer canthus. Signals were amplified and digitized at 10 kHz (BIOPAC system, MP150), and stored for off-line analysis. The HBR was elicited by delivering transcutaneous electrical stimuli to the median nerve, using a surface bipolar electrode attached with a Velcro strap (constant current square-wave pulses; DS7A, Digitimer). Firstly, the stimulus intensity able to elicit clear BR was calibrated per participant, by increasing the stimulus intensity until a clear and stable HBR was observed in at list five consecutive trials, or participant refused further intensity increase (mean stimulus intensities, 33.12 ± 11.67 mA; range, 20–60 mA). The stimulus duration was 200 μs and to minimize stimulus habituation the inter-stimulus interval was ~30 s, following prior method^20^,^21^,^22^,^26^.

### Procedure

Participants were seating comfortable and before starting the experiment they were instructed to keep the hand still and to wait the stimuli in two different positions: far (i.e. the forearm resting on a pillow with the hand at a distance of ~60 cm from the face) and near (i.e. the hand at the distance of ~4 cm from the ipsilateral side of their face). E+ patients were informed that, in the affected body-side, they would be helped by the experimenter to execute the postural manipulation. Throughout the experiment, during the EMG recording, participants had to keep their gaze on a fixation cross in front of them (at a distance of about 50 cm). At the beginning of the experiment participants were informed that stimuli could be delivered to either hand, but crucially, they did not know in advance which of the two hands would be stimulated on each trial. Finally, they were asked to report in which of the two hands (left or right) and in which position (far or near) they felt the stimulus. Note that, in the alien conditions, healthy controls, E− patient and E+ patients in the intact body-side always referred to feel the stimuli in the far position; only E+ patients, in the affected body-side, referred to feel the stimuli in the near position (where the alien hand was located). See the accuracy percentage of stimuli detection in Preliminary results.

### Embodiment Evaluation during the task

In order to evaluate the presence/absence of the pathological embodiment during the task, at the end of each block, we addressed the following questions:

Did you perform the task with your left/right hand?

In intact body-side session, both E− patient and all E+ patients positively answered this question only at the end of the own blocks (where the own hand undertook the postural manipulation), while, at the end of the alien blocks (where the alien hand undertook the postural manipulation), they gave negative answers, correctly acknowledging that the alien hand did not belonged to themselves.

Crucially, in the affected body-side session, all E+ patients positively answered this question at the end of both the own and the alien block, claiming that the alien hand undertaking the postural manipulation was their own hand. On the contrary, E− patient positively answered this question only at the end of the own block, while, as in the intact body side, at the end of the alien blocks (where the alien hand undertook the postural manipulation), he gave negative answers, and correctly acknowledging that the alien hand did not belonged to him.

### Preliminary studies

In designing the experiment, we had to take into account the E+ patients’ neurological characteristics. The pathological embodiment was usually associated with contralesional upper limb motor deficits (i.e. hemiplegia). This implied two main consequences on our experimental design. First of all, E+ patients could not autonomously perform the postural manipulation in the contralesional (affected) body-side, therefore the patients’ hand could be kept close to their face only by means of the experimenter’s help. Secondly, in the literature it has been described a suppression of the R2 blink reflex after stimulation of the affected side in patients with hemiplegia, and this suppression was attributed to the decreased cortical-reticular drive^37^. According to these findings, it was likely to expect no HBR responses by delivering electrical stimuli on the patients’ affected hand.

In order to face the first issue and rule out the possibility that the experimenter’s help was the factor determining the HBR modulation observed in the main study, we performed a preliminary experiment in ten normal subjects (4 males and 6 females, mean age ± standard deviation: 24.1 ± 1.19). We aimed to test whether the HBR magnitude was enhanced, compared to the baseline-far position, irrespective of whether the hand bringing the threat was actively (active-near condition) or passively (i.e. by means of the experimenter’s help, passive-near condition) kept close to the face. No difference between the active-near and the passive-near condition was found (p = 0.62, paired T-test, two-tailed). A similar increase with respect to the baseline-far position was found when the hand was actively kept close to the face (active-near condition) or when it was undertaken by an external support (passive-near condition) (baseline-far: 3.7 ± 1.3; active-near: 5.1 ± 2.3; +37.14 ± 26.6%; passive-near: 4.94 ± 1.82; +35.26 ± 29.8%; active-near vs. baseline-far: p = 0.0048; passive-near vs. baseline-far: p = 0.0013; paired T-test, two-tailed). This means that the presence of the experimenter’s help was not a significant factor by itself in the HBR modulation. Therefore, according to these preliminary results, we assumed that, in the main experiment, performing the postural manipulation by means of the experimenter’s help would not interfere with the HBR modulation.

In order to face the second issue, related the suppression of the R2 blink reflex after stimulation of the hemiplegic patients’ affected side, we took advantage from an experimental design, recently described by Sambo & co^20^. In their study, the electrical stimuli were delivered, with equal probability, to either the hand undergoing the postural far/near manipulation (‘moving hand’) or the other hand (‘non-moving hand’, always kept in the far position). Their results showed that a significant HBR increase was present when the stimuli were delivered to both the moving and the non-moving hand, although for the non-moving hand the HBR enhancement was lower. Based on this study, we decided to deliver stimuli to both the hand undergoing the postural manipulation and the other hand (in a pseudorandom order, with no more than two consecutive stimuli to the same hand). In this way, when the postural manipulation was performed in the patient’s affected body-side, we could have a chance to see an HBR enhancement evoked by the stimulation of the intact (non-moving) hand.

### Experimental design

We employed a between-subject experimental design, where both patients and healthy controls were tested in three experimental conditions. In the ‘baseline-far’ condition, the participant’s stimulated hand was located outside the DPPS of the face, laid down on the table. In the ‘own-hand-near’ condition the participants’ stimulated hand was located close to the face inside the DPPS of the face (note that the patients’ paralyzed hand was passively kept close to the face by means of the examiner’s help). In the ‘alien-hand-near’ condition another person’s hand was located inside the participant’s DPPS, while the participant’s stimulated hand was located in the far position (see Fig. 1). The experiment consisted of two sessions (recorded in two different days), according to the body-side where the postural manipulation was performed: the right/intact and the left/affected body-side. Each session consisted of two blocks for each condition administered in a counterbalanced order (own-hand, alien-hand, alien hand, own-hand). Each block consisted of a total of 16 electrical stimuli, delivered as follows: 8 stimuli were ipsilateral to the postural manipulation side and the other 8 contralateral (4 in the baseline-far position and 4 in the near position in alternating trials).

### Preliminary analysis and preliminary results

The patients tested here had problems in reporting the electrical stimuli when applied on their affected wrist (accuracy percentage in reporting the stimuli: E+1 = affected 10%; intact 100%; E+2 = affected 30%; intact 100%; E+3 = affected 35%; intact 100%; E+4 = affected 90%; intact 100%; E+5 = affected 36%; intact 100%; E−1 = affected 40%; intact 100%). As expected according to the literature^37^, no HBR response was recorded after the stimulation of the patients’ affected hand. Thus, in patients, only the eye blink elicited by the stimulation of the intact hand was considered for the further analysis. In summary, in the intact body-side session, we considered the responses that were elicited by stimuli ipsilateral to the postural manipulation; on the contrary, in the affected body-side session, we considered the responses that were elicited by stimuli contralateral to the postural manipulation.

In healthy controls, we replicated the results of the Sambo & co.^20^ study. We performed a 2 × 2 repeated measure ANOVA on the row AUC data with two within-subjects factors: hand position (two levels: far; near) and stimulation-side (two levels: moving hand; no-moving hand). We found a significant main effect of the factor ‘hand position’ (F_(1,9)_ = 32.86, p = 0.0003) and a significant interaction between the factor ‘hand position’ and ‘stimulation-side’ (F_(1,9)_ = 7.11, p = 0.02). Crucially, although the HBR enhancement was stronger when the stimulated hand is the one undertaking the postural manipulation (moving hand far: 9.41 ± 5.14; moving hand near: 13.94 ± 6.96; p = 0.0001, at post hoc comparison), a significant enhancement was found also when the no-moving hand undertook the postural manipulation (no-moving hand far: 10.58 ± 7.02; no-moving hand near: 12.72 ± 7.27; p = 0.008).

### Data preprocessing

EMG pre-processing and analysis were completed using MatLab (http://www.mathworks.com) and Letswave (http://nocions.webnode.com) software^54^. EMG signals from each participant were high-pass filtered (55 Hz), full-wave rectified, then averaged across ipsilateral and contralateral recording side. HBR responses were averaged separately according to condition (baseline-far; own-hand-near; alien-hand-near) either for the left/affected or right/intact body-side, resulting in six waveforms for each subject. We extracted and measured the area under the curve (AUC) of the average HBR waveform for each subject and condition. In order to perform group analysis, data were normalized within subjects using Z-scores [(x-mean)/standard deviation]^12^.

### Data analysis

According to the literature^37^, preliminary results showed that, in patients, the HBR response was recorded only after the stimulation of the intact body-side. Thus, in the between group analysis, for the comparison between controls and the patients’ intact body-side, in both groups, only responses elicited by the stimuli ipsilateral to the postural manipulation were considered. On the contrary, for the comparison between controls and the patients’ affected body-side, in both groups, only responses elicited by the stimuli contralateral to the postural manipulation were considered.

In the analysis, we used as dependent variable the AUC data (normalized in z-scores) for the ‘own-hand-near’ and the ‘alien-hand-near’ conditions expressed as a difference respect to the ‘baseline-far’ condition. The obtained values, representing the response enhancement in the near conditions, were entered in a 2 × 2 × 2 repeated measure ANOVA with one between-subjects factor, ‘group’ (controls; E+ patients), and two within-subjects factors, ‘body-side’ (right/intact; left/affected) and ‘hand ownership’ (own; alien).

We verified the normal distribution of the residuals with a Shapiro-Wilk test: W = 0.96963; p < 0.16902. We checked for the equivalence of variance of Patients’ and healthy controls’ HBR responses in all conditions by means of an F-test for the equivalence of variance. It never reached the significance level (patients’ intact side vs control p = 0.84; patients’ affected side vs control p = 0.74), suggesting that the equivalence of variance can be assumed and the ANOVA run properly.

Finally, in order to compare the different HBR enhancement between E− patient and both E+ patients and control subjects group, single-subject analysis were performed by means of the Crawford test^27^. As for the main analysis, we used as dependent variable the AUC data for the ‘own-hand-near’ and the ‘alien-hand-near’ conditions expressed as a difference respect to the ‘baseline-far’ condition.

## Additional Information

**How to cite this article**: Fossataro, C. *et al*. Bodily ownership modulation in defensive responses: physiological evidence in brain-damaged patients with pathological embodiment of other’s body parts. *Sci. Rep.* **6**, 27737; doi: 10.1038/srep27737 (2016).

## Figures and Tables

**Table 1:** Patients’ demographic and clinical data.

Patients’ neuropsychological assessment	E+1	E+2	E+3	E+4	E+5	E−1
Sex	F	F	M	M	F	M
Education	5	13	8	5	8	10
Etiology	E	I	I	E	I	I
Lesion Side	LH	RH	RH	RH	RH	RH
Month from onset	6	2	2	2	2	4
General cognitive impairment		−	−	−	−	−
Visual Field Defect	0–0	0–0	0–0	0–0	0–0	0–0
Hemiplegia (HP)	3	3	3	3	3	3
Hemianaesthesia (HA)	3	3	3	1	3	2
Anosognosia for HP	0	0	0	0	0	0
Anosognosia for HA	0	0	3	/	3	0
Neglect	−	−	−	−	+	−
Personal Neglect	−	+	+	+	−	−
Somatoparaphrenia	−	−	−	−	−	−

Presence/absence of embodiment (E+/E−) of the experimenter’s arm. Sex: M = Male, F = Female. Education: years of school. Etiology: H = hemorrhage; I = ischemia. Lesion Side: RH = Right Hemisphere; LH = Left Hemisphere. Months from onset: number of months between the onset of the disease and the first assessment. For visual field defect (the two values refer to the upper and lower visual quadrants, respectively), hemiplegia, anosognosia for hemiplegia and hemianesthesia scores were ranged from normal (0) to severe defects (3)^48^. General cognitive impairment (− = no deficit; + = presence of deficit): MOCA cut off ≥ 14.5/30; MMSE cut off ≥ 24/30. Neglect (− = no deficit; + = presence of deficit;): BIT, conventional subtests cut-off ≥ 129/146; BIT behavioral subtest cut-off ≥ 67/81; DILLER cut-off omissions l–r ≥ 5. Personal neglect (− = no deficit; + = presence of deficit;): FLUFF cut off omissions L ≤ 2. The presence/absence of somatoparaphrenia was evaluated according to Fotopoulou *et al*.^49^.
